# Epigenetic Events in Liver Cancer Resulting From Alcoholic Liver Disease

**DOI:** 10.35946/arcr.v35.1.07

**Published:** 2013

**Authors:** Samuel W. French

**Affiliations:** **Samuel W. French, M.D.,***is a distinguished professor in the Department of Pathology at the Harbor UCLA Medical Center in Torrance, California.*

**Keywords:** Alcohol consumption, alcohol abuse, chronic alcohol use, alcoholic liver disease, ethanol metabolism, alcoholic liver disease, liver cancer, hepatocellular carcinoma, epigenetics, epigenetic mechanisms, DNA methylation, histone methylation, stem cells, micro RNAs

## Abstract

Epigenetic mechanisms play an extensive role in the development of liver cancer (i.e., hepatocellular carcinoma [HCC]) associated with alcoholic liver disease (ALD) as well as in liver disease associated with other conditions. For example, epigenetic mechanisms, such as changes in the methylation and/or acetylation pattern of certain DNA regions or of the histone proteins around which the DNA is wrapped, contribute to the reversion of normal liver cells into progenitor and stem cells that can develop into HCC. Chronic exposure to beverage alcohol (i.e., ethanol) can induce all of these epigenetic changes. Thus, ethanol metabolism results in the formation of compounds that can cause changes in DNA methylation and interfere with other components of the normal processes regulating DNA methylation. Alcohol exposure also can alter histone acetylation/deacetylation and methylation patterns through a variety of mechanisms and signaling pathways. Alcohol also acts indirectly on another molecule called toll-like receptor 4 (TLR4) that is a key component in a crucial regulatory pathway in the cells and whose dysregulation is involved in the development of HCC. Finally, alcohol use regulates an epigenetic mechanism involving small molecules called miRNAs that control transcriptional events and the expression of genes important to ALD.

The molecular pathogenesis of liver cancer (i.e., hepatocellular carcinoma [HCC]) is a multistep process that involves both genetic changes, such as chromosomal abnormalities and mutations of the DNA sequence (i.e., somatic mutations), and epigenetic mechanisms, such as chemical modifications of the DNA and the histone proteins around which the DNA is wrapped to form the chromosomes, microRNA post-transcriptional regulators, and changes in various signaling pathways (Wong et al. 2010). This review will focus on the epigenetic phenomena that contribute to the pathogenesis of HCC resulting from alcoholic liver disease (ALD).

## Does ALD Lead to HCC Formation?

According to some studies, ALD is the most common cause of HCC, accounting for approximately one-third of all HCC cases (Morgan et al. 2004). Chronic alcohol use of greater than 80 g/day (or approximately three standard drinks or more per day) for more than 10 years increases the risk for HCC approximately fivefold. In patients with decompensated alcoholic cirrhosis, in whom the liver damage is so extensive that the functional portions of the organ can no longer compensate for the damaged ones, the risk of developing HCC approaches 1 percent per year, and this risk does not decrease with abstinence (Morgan et al. 2004). However, HCC also can occur in patients with noncirrhotic ALD. Finally, HCC is more likely to develop 1 to 10 years after the cessation of drinking by ALD patients. Therefore, HCC in these patients is not directly caused by alcohol consumption (Donato et al. 2002).

Alcohol abuse also has synergistic effects with other risk factors for the development of HCC, such as infection with hepatitis B virus (HBV) or hepatitis C virus (HCV), diabetes, and obesity (Hassan et al. 2002; Loomb et al. 2010; Morgan et al. 2004). For example, studies in Italy (Tagger et al. 1999) and the United States (Hassan et al. 2002) found that in patients with HCV infection, alcohol consumption over 80 g/day increased the odds ratio of developing HCC by 7.3 and 4.5, respectively. Likewise, a study conducted in Africa (Mohamed et al. 1992) determined a synergism between HBV and alcohol consumption over 80 g/day in the development of HCC (odds ratio of 4.4).

## What Do ALD, HCV, and HBV Have in Common?

The livers of patients who develop HCCs commonly are cirrhotic. Moreover, they often contain molecules (i.e., markers) indicating that the cells undergo changes in their structure and function to a less specialized (i.e., less differentiated) state. These progenitor/stem cell markers mainly are found in the cirrhotic portion of the liver and in the regions where the HCC develops. These changes and markers have been observed in the livers of patients developing HCC associated with ALD, HBV, and HCV (Oliva et al. 2010). The reversion of normal liver cells (i.e., hepatocytes) into progenitor and stem cells is caused by epigenetic mechanisms. For example, during the development of the progenitor and stem cells, changes occur in the expression of several genes that result from the addition of too many or fewer-than-normal methyl groups to the DNA (i.e., DNA hyper- and hypomethylation, respectively). This alteration of methylation patterns results in an epigenetic reprogramming of the cells (Alison et al. 2009; Collas 2009; Iacobuzio-Donohue 2009; Ohm and Baylin 2009; Richly et al. 2010; Sasaki 2006; Sawan et al. 2008). In addition, modification (i.e., methylation and the addition of acetyl groups [acetylation]) of the histone proteins play roles in the epigenetic modification of progenitor and stem cells that underlies the transformation into cancer cells (i.e., a carcinoma) (Iacobuzio-Donohue 2009). Alcohol excess can induce all of these epigenetic changes that contribute to the transformation of hepatocytes into progenitor or stem cells.

## How Does Alcohol Generate Epigenetic Changes?

### DNA Methylation

One step in the metabolism of beverage alcohol (i.e., ethanol) in the liver is the oxidation of ethanol by a molecule called cytochrome P450 2E1 (CYP2E1). During this reaction, highly reactive, oxygen-containing molecules (i.e., reactive oxygen species [ROS]) are generated (Bardag-Gorce et al. 2006). ROS are among the most potent agents and conditions that can alter methylation patterns in the liver, including DNA methylation. Thus, oxidative DNA damage caused by ROS, such as the formation of an abnormal variant of the DNA building block (i.e., nucleotide) deoxyguanine called 8-oxyguanine (8-OHdG), can result in a decrease in methylated DNA during DNA repair (Weitzman et al. 1994). 8-OHdG can be incorporated into DNA regions rich in the nucleotides cytosine and guanosine (i.e., CpG islands) in which the cytosine residues frequently are methylated. Incorporation of 8-OHdG into such CpG islands inhibits the methylation of adjacent cytosine residues by enzymes called methyl transferases, resulting in hypomethylation. Also, 8-OHdG formation can interfere with the normal function of DNA methyl transferases and prevent DNA re-methylation (Sagaki 2006). The relationship between alcohol, ROS formation, and DNA damage was demonstrated by studies in cultured liver cells (i.e., HepG2 cells) that were genetically modified to produce excessive levels of CYP2E1. When these cells were incubated with ethanol, ROS-induced DNA damage occurred as indicated by the formation of 8-OHdG (Bardag-Gorce 2006).

Ethanol also interferes with the metabolism of the amino acid methionine into a compound called S-adenosyl-methionine (SAMe) by several different methyl transferase reactions. SAMe, in turn, is needed as the methyl-group donor for many methylation reactions and is converted into S-adenosyl-homocysteine (SAH). Ethanol inhibits methionine adenosyl transferase, which converts methionine into SAMe, as well as enzymes that help regenerate methionine (i.e., betaine homocysteine methyltransferase and methionine synthase) (Seitz and Sticke 2007). This was shown in rodent models of ALD, where ethanol feeding decreased the SAMe/SAH ratio in the liver (Esfandari et al. 2010). The net effect of all these alcohol-induced reductions in methyl transferase activity is to reduce the synthesis of SAMe, which in turn leads to a decrease in DNA methylation.

The significance of reduced SAMe production in the development of HCC is supported by findings that SAMe feeding can inhibit tumor formation (Hitchler and Domann 2009). Furthermore, studies found that the SAMe content and the SAMe/SAH ratio were decreased in tissue regions that showed some damage but had not yet turned into cancer cells (i.e., in preneoplastic lesions). SAMe feeding blocked the transformation of these preneoplastic lesions into HCCs because it promoted global DNA methylation. Moreover, SAMe administration inhibited the expression of certain cancer-inducing genes (i.e., proto-oncogenes) called *c-myc, c-Ha-ras*, and *c-K-ras*, because the SAMe supplementation allowed for the methylation (and thus blockage) of the regulatory regions (i.e., promoters) for those genes. The potential role of SAMe in preventing tumor formation and survival also was supported by an in vitro study demonstrating that SAMe decreased the survival of a type of liver cell tumor Hepa 1–6 in a dose-dependent manner (Oliva et al. 2012). Finally, SAMe treatment prevented cultured liver tumor cells (i.e., H411e cells) from forming a tumor in a model of laboratory rats (Lu et al. 2009).

### Histone Modifications

Histones, which exist in numerous variants, regulate gene expression, with the level of gene expression depending on the modifications that the histones undergo. These modifications may include methylation, acetylation, the addition of phosphate groups (i.e., phosphorylation), or the addition of a molecule called ubiquitin (i.e., ubiquitination). These modifications also can have an impact on tumor development. For example, the removal of acetyl groups (i.e., deacetylation) as well as hypermethylation is linked to the inactivation (i.e., silencing) of genes that can help repress tumor formation (i.e., tumor suppression genes) and as a result may promote tumor development (i.e., carcinogenesis). Thus, some cancers exhibit CpG island hypermethylation in combination with multiple histone modifications, such as deacetylation of histones H3 and H4, methylation of histone H3K9, trimethylation of histone H3K27, and a loss of trimethylation of histone H3K4 (Hamilton 2010).

#### Histone Acetylation

Alcohol exposure can alter histone acetylation and methylation patterns. Researchers have investigated these effects in rats that chronically were fed alcohol through a tube into the stomach (i.e., intragastric tube feeding). These studies identified several alterations in histone methylation and acetylation that correlated with the changes seen in HCCs. Thus, alcohol-treated animals showed increased acetylation of histone H3K18 (Bardag-Gorce et al. 2009) and histone H3K9 (Bardag-Gorce et al. 2007). Furthermore, the levels of several proteins (i.e., phospho c-Jun, phospho AKT threonine 308, p38, pERK, and phospho-SAPK/JNK) in the nucleus of HCC cells were reduced whereas the nuclear levels of a molecule called β-catenin were increased. (For a list of the genes and proteins and their main functions, see table 1.) An increase in β-catenin in the nucleus of hepatocytes indicates activation of a signaling pathway, known as the canonical WNT/β-catenin pathway,^1^ that can be involved in tumor formation. This often is seen in HCCs related to ALD and HBV and HCV infection and leads to abnormal cell proliferation and survival (Hamilton 2010). The chronically alcohol-fed rats also had increased levels of an enzyme called histone acetyltransferase (HAT) p300, which is responsible for histone acetylation (Bardag-Gorce et al. 2007). This increase could explain the increased histone H3K4 and H3K9 acetylation, which, in turn, globally activates gene expression (Bardag-Gorce 2009). Simultaneously, the levels of a deacetylase (i.e., SIRT1) also were increased in the alcohol-fed animals (Bardag-Gorce et al. 2007; 2009). This change was accompanied by alterations in the levels of several other molecules, including increases in RARb and peroxisome proliferator– activated receptor (PPAR) C coactivator 1α (PGC1α) expression and a decrease in PPARγ expression.

**Figure 1:**
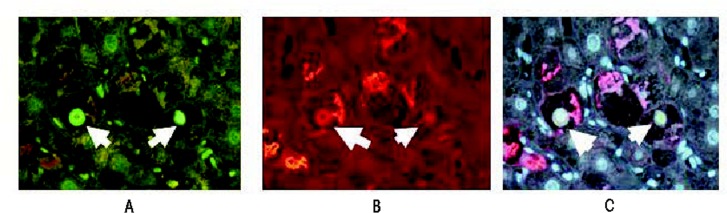
Histone deacetylase 1 (HDAC1) is upregulated in the nuclei of liver cells (i.e., hepatocytes) that form Mallory-Denk bodies (MDBs), which are indicative of liver damage. The image shown is from a liver biopsy from a patient with alcoholic hepatitis. The liver section was IHC double stained for HDAC1 (green nuclei arrows) **(A)**, ubiquitin to identify cells with MDBs (red, arrows) **(B),** and tricolor **(C)**. Magnification: ×350.

The increase in HAT p300 levels observed in chronically alcohol-fed rats also could lead to an increase in a signaling molecule called p21WAF1/C, p1 (p21) through several direct and indirect mechanisms^2^ (Fang et al. 2007). p21 and a related protein called p27 are enzyme complexes that can mediate the phosphorylation of certain other proteins (i.e, protein kinase complexes) and which cause delays in the cycle progression at various stages of the cell cycle, thereby preventing the cells from dividing and multiplying normally. This leads to cell-cycle arrest, genetic instability, programmed cell death (i.e., apoptosis), and oncogenic effects (Abbas and Dutta 2011; Serres et al. 2011, 2012). p21 expression is regulated by histone acetylation, with greater acetylation promoting p21 expression. This process is regulated by a protein complex that is associated with the p21 promoter and which includes an enzyme called histone deacetylase-1 (HDAC1) that reduces acetylation (Dokmanovic et al. 2007) and, thus, p21 expression. Agents that promote acetylation by inhibiting deacetylation (i.e., HDAC inhibitors) accordingly also induce p21 expression, causing cell-cycle arrest (Dokmanovic et al. 2007; Gui et al. 2004). For this reason, HDAC inhibitors are used to treat cancers (Drummond et al. 2005). Liver cells that show signs of ALD—that is, which form Mallory-Denk bodies (MDBs)^3^—show increased HDAC1 levels in their nuclei compared with adjacent normal hepatocytes (see figure 1) (French et al. 2010). The HDAC inhibitor trichstatin A inhibited formation of MDBs in cell cultures from the livers of drug-primed mice (Oliva et al. 2008), indicating that these agents also may be able to prevent the development of liver disease.

The induction of p21 by alcohol abuse may explain why HCC more often only occurs after the patient has stopped drinking. As mentioned above, alcohol consumption induces p21 expression, causing the cell-cycle arrest. After prolonged abstinence, this induction no longer persists in the liver, eliminating the cell cycle arrest and promoting cell multiplication and, thus, tumor formation.

**Figure 2:**
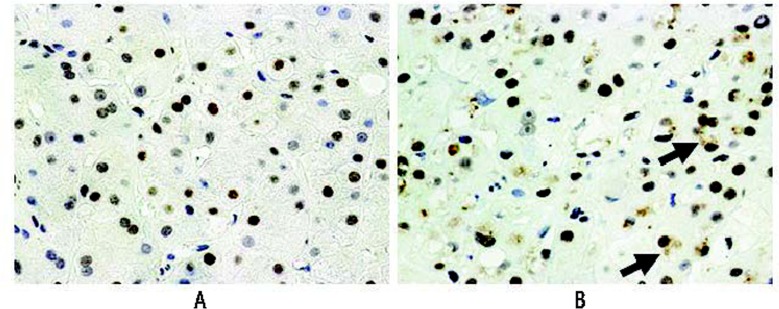
The signaling molecule p27 is upregulated in the nuclei of liver cells (i.e., hepatocytes) in a liver biopsy from two patients with alcoholic hepatitis. The livers were stained with an immunoperoxidase-labeled antibody that recognizes p27. The hepatocyte nuclei positive for p27 appear brown; those that are negative for p27 appear blue. **(A** and **B)** Most of the nuclei stained positive. **(B)** The Mallory-Denk bodies (MDBs) also stained brown (arrows), indicating that p27 also is sequestered in the MDBs. Magnification ×520.

The role of p21 and p27 in HCC also is supported by studies showing that both proteins are overexpressed in alcoholic hepatitis and in rats chronically fed ethanol (Crary and Albrecht 1998; French et al. 2012; Koteish et al. 2007) For example, immunohisto-chemical studies of liver samples from patients with alcoholic hepatitis found that many of the cells were positive for p27 (see figure 2), and additional analyses indicated that cell-cycle progression was blocked in these cells as indicated by low numbers of nuclei showing expression of (i.e., positive for) a protein called ki-67 (French et al. 2012). Another study demonstrated that both p21 and p27 overexpression inhibit the regeneration of the liver in rats whose liver had been partially removed (Koteish et al. 2007).

#### Histone Methylation

The levels of methylated histone H3K4 (H3K4me2), as well as histone H3K27 (H3K27me3), are increased in the nuclei of liver cells from rats fed ethanol intragastrically for 1 month (Bardag-Gorce et al. 2009), as demonstrated by intense nuclear staining in immunohisto-chemical analyses of liver samples. H3K4me2 is associated with active transcription, which seems to have beneficial effects. In particular, the combination of H3K4me2 with acetylated histone H3K18, which is seen in the alcohol-fed rats, would correlate with an improved prognosis in cancers. A loss of H3K4me2 impairs the body’s ability to control DNA damage in cancer because it increases the risk of mutations and, consequently, cancer development (Lennartsson and Ekwall 2009).

Whereas histone acetylation is a highly dynamic process, modification of histones by methylation of one or more lysine amino acids (i.e., mono-, di-, and trimethylation) is thought to be a more lasting change that forms a “cellular memory.” Methylation is performed by enzymes known as methyl transferases, and the activity of these enzymes may be specific to certain histones. The enzymes that generate persistent methylation patterns and other histone modifications are known as histone code writers.^4^ One such enzyme called EZH2 has intrinsic histone H3K27 methyl transferase activity; it assembles into a multiprotein complex called polycomb repressive complex 2 (PRC2) that, together with another protein complex (i.e., PRC1), maintains a state of transcriptional repression and plays an important role in gene silencing (Muntean and Hess 2009). One role of EZH2/H3K27me3 is to target PRCs to sites of transcriptional regulation and DNA replication. The latter provides the means of perpetuating the characteristics (i.e., phenotypes) of the dividing cell to the daughter cells.

**Figure 3:**
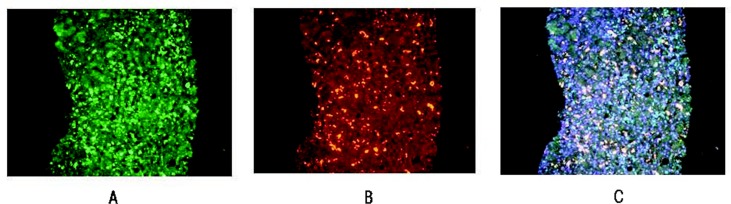
These images show a double-immunostained liver biopsy from a patient with alcoholic hepatitis where most of the hepatocytes had formed Mallory-Denk bodies (MDBs). The MDBs stained positive for **(A)** pEZHZ (green), **(B)** ubiquitin (red), and **(C)** merged (yellow), indicating that the pEZH2 colocalized in the MDBs. Magnification ×350.

The gene-silencing pathway mediated by H3K27 methylation is linked to the second major silencing pathway (i.e., DNA methylation) via a deacetylase called SIRT 1 that is recruited by the PRC2 complex and contributes to gene silencing (Muntean and Hess 2009). SIRT 1 levels are increased in the alcohol intragastric tube-feeding rat model cited above. In contrast, when SIRT 1 activity is decreased, EZH2 levels increase, which enhances the EZH2-mediated repression of target genes (Lu et al. 2011). Upregulation of EZH2 expression in tumors appears to correlate with disease progression by maintaining a stem cell-like phenotype. Overexpression of EZH2 can lead to cancer progression mediated by deregulation of epigenetic mechanisms (Muntean and Hess 2009). However, EZH2 levels do not seem to be affected by alcohol and other factors that can induce liver damage. For example, EZH2 levels were not changed in mice that exhibited a precursor stage to HCC (as characterized by the presence of balloon cells and MDBs) after drug treatment, in liver biopsies of patients with alcoholic hepatitis, or in MDB-forming HCCs (French et al. 2012) (see figure 3). However, in all three cases there were increases in a modified form of EZH2 (i.e., phosphorylated EZH2 [pEZH2]), which is degraded more rapidly in the cells than unmodified EZH2 and is located in the MDBs as demonstrated by immunohisto-chemistry. This degradation of pEZH2 occurs at cell components called proteasomes. However, proteasomes are inhibited by ethanol excess; as a result, pEZH2 levels are increased in MDBs. Moreover, in all three cases, the levels of H3K27me3 were reduced in the nuclei of the damaged liver cells (i.e., cells that were ballooned or formed MDBs) compared with neighboring normal liver cells as shown by different experimental approaches (Bardag-Gorce et al. 2010; French et al. 2012). Paradoxically, when tumors form, they overexpress EZH2. High expression of EZH2 in tumors is associated with poor survival (Gieni and Hendzel 2009). Thus, EZH2 overexpression represses expression of the product of a tumor suppressor gene called E cadherin that causes cells to stick to each other. Accordingly, loss of E cadherin expression by tumor cells may cause loss of cell cohesion, which would promote metastasis and thus a more unfavorable prognosis.

## How Are Stem Cells Converted to Cancer Stem Cells in ALD?

**Figure 4:**
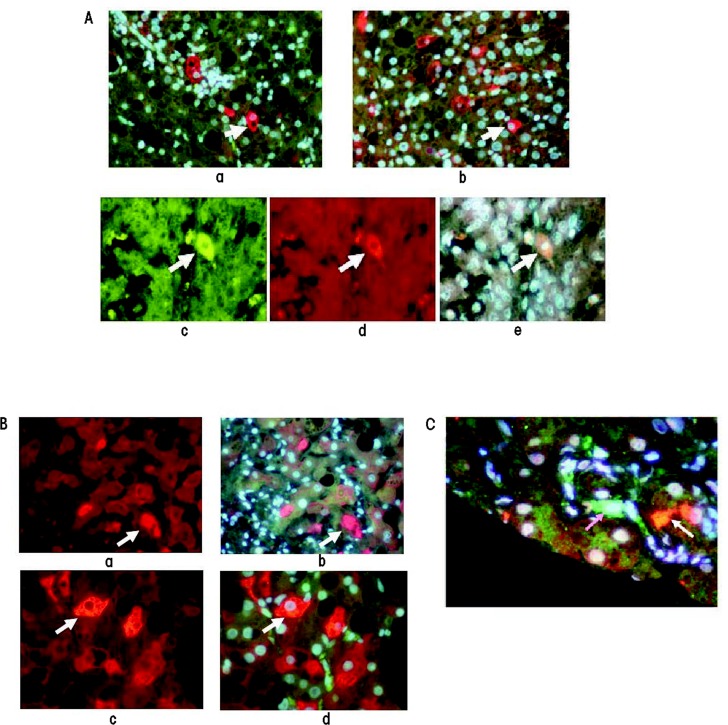
Analysis of different marker proteins in stem cell/progenitor cells located in the livers of patients with alcoholic liver disease with cirrhosis and associated hepatocellular carcinoma (HCC). **(A)** Liver cirrhosis and HCC samples stained for both YAP-1 (green) and IGF2bp3 (red). a) Cirrhosis; b) HCC (magnification ×350); c) HCC; d) HCC; e) Tricolor image merged from c and d (magnification ×525). **(B)**, a and b) Liver cirrhosis sample double stained for Nanog (green) and SOX2 (red). Note the Mallory-Denk bodies (MDBs) (arrow) stain positive for SOX 2. c and d) Liver cells stained for Yap 1 (green) and SOX 2 (red). The liver cells/progenitor cells stain positive for SOX2 (arrows). Magnification ×780. **(C)** Liver sample from a patient with alcoholic hepatitis double stained for the Nanog protein (green) and ubiquitin (red). The stem cell stains positive for Nanog (pink arrow) and an MDB stained positive for ubiquitin (white arrow). Magnification ×780.

In individuals with HCC associated with ALD, focal progenitor cell/stem cell formation occurs both in portions of the liver that show cirrhosis and in the HCC cells as indicated by the expression of certain proteins (i.e., Nanog, Yapi-1, Igf2bp, and Sox2) (see figure 4 A, B, C,). This raises the question whether the liver cells that are transformed into progenitor cells/stem cells in the cirrhotic liver subsequently are transformed in a second step into cancer stem cells in HCC. One of the regulatory molecules involved in this process is called Nanog. It is a transcription factor that is thought to play a crucial role in the self-renewal of embryonic stem cells and helps them maintain their ability to subsequently differentiate into numerous other cell types. Cancer stem cells can express both EZH2/H3k27me3 and Nanog, and the epigenetic balance between these factors determines the further fate of the cells. When the levels of Nanog are high and those of EZH2/H3K27me3 are low, the cells exhibit self-renewal activity—that is, they multiply and a tumor can develop. Paradoxically, when the reverse is true, the cancer stem cells differentiate into cells that no longer proliferate (Villasante et al. 2011). Also, EZH2-mediated epigenetic silencing of tumor suppressor genes leads to the activation of the WNT/β-catenin signaling pathway mentioned earlier, which culminates in the proliferation of HCC cells (Cheng et al. 2011). EZH2 overexpression occurs in many different cancers, where it acts as a classical oncogene that can promote tumor formation by silencing several tumor suppressor genes, such as E cadherin. These suppressor genes play a role for both tumor cells and cancer stem cells (Crea 2011).

### What Role Do Epigenetic Changes in TLR4 Play in Stem Cell Transformation?

Another molecule that is involved in the epigenetic mechanisms contributing to ALD-related HCC and which helps regulate the activity of Nanog is called toll-like receptor 4 (TLR4). Studies in a certain line of genetically modified mice (i.e., HCV core transgenic mice) that were chronically fed alcohol found that TLR4 activation leads to up regulation of Nanog in stem cells (Machida et al. 2009). This TLR4–Nanog pathway promotes the development of liver tumors induced by a variety of factors, including alcohol, diabetes, and HCV (Machida et al. 2012). The activation of TLR4 is regulated both at the transcriptional level (i.e., via molecules called lipopoly-saccharides [LPS]) and at the epigenetic level (i.e., via acetylation of histones and methylation of DNA). For instance, increased methylation of regulatory DNA regions in front of the gene encoding TLR4 was found in embryonic stem cells. Moreover, increased methylation suppressed TLR promoter activity in reporter gene assays (Zampetaki et al. 2006). In addition, other assays (i.e., CHiP assays) in embryonic stem cells demonstrated that histones H3 and H4 had lower-than-normal acetylation levels (i.e., were hypoacetylated) in the TLR promoter region. Treatment with inhibitors of DNA methylation or deacetylase partially relieved repression of the TLR4 gene and increased its responsiveness to LPS (Zampetaki et al. 2006). The combined inhibition of DNA methylation and histone deacetylase activity leads to a robust induction of TLR4 with return of LPS responsiveness.

Rats fed ethanol intragastrically for 1 month had increased levels of TLR4 and another molecule called MyD88 in their livers. This effect could be prevented by feeding the animals SAMe together with the alcohol, which, as mentioned earlier, is required for methylation. These findings indicate that methylation can prevent the alcohol-induced changes in TLR4 and MyD88 levels (Oliva et al. 2012). Similar changes in TLR4 expression and protein levels were found in mice that developed liver tumors after being fed a compound called diethyl 1,4-dihydro-2,4,6-trimethyl-3,5pyridinedicarboxylate (DDC). Again, the changes could be prevented by also feeding the animals SAMe (Bardag-Gorce et al. 2010). More detailed analyses determined reductions in the levels of H3k27me3 that also could be prevented by SAMe feeding. These findings indicate that DDC feeding causes histone demethylation, which in turn results in increased TLR4 expression (Bardag-Gorce et al. 2010). In fact, the mice exhibited numerous epigenetic changes of histone methylation and acetylation (Bardag-Gorce et al. 2008), as well as DNA methylation of the gene encoding interleukin 12A (Oliva and French 2012).

Researchers also have studied the TLR4-Nanog pathway in another line of genetically modified mice (i.e., HCV Ns5a transgenic mice) that were fed alcohol; under these conditions, liver tumors form in the animals that contain cancer stem cells (Machida et al. 2012). During this process, cells that normally differentiate into hepatocytes (i.e., hepatic stem cells) are transformed into tumor-initiating stem-like cells (TISCs), which then may develop further into cancer cells and cause tumor formation in other tissues. For example, TISC cells isolated from alcoholic patients induced tumor formation in cultured tissues (i.e., in vitro) and after transplantation into laboratory animals (i.e., in a xenograft model). The role of TLR4 and Nanog in this process was demonstrated by findings that when TLR4 or Nanog were silenced, the tumor-initiating properties of the TISCs were attenuated. Further studies found that Nanog upregulated the expression of two genes encoding molecules called Yap 1 and activator Igf2bp3, which in turn inhibited transforming growth factor-β signaling in the TISCs. Transforming growth factor-β signaling inhibits the growth of liver cells; accordingly, its inhibition would favor the proliferation of TISCs to form liver tumors. These observations suggest that TLR4 may be a universal protooncogene that is responsible for the development of TLR/Nanog-dependent TISCs. By staining tissue samples with specific markers researchers demonstrated that TISCs can be found in patients with cirrhosis and HCCs caused by alcoholism as well as by nonalcoholic hepatitis and HBV or HCV infection (Bardag-Gorce et al. 2008; French et al. 2011; Oliva et al. 2010) (figures 4A–C).

**Figure 5:**
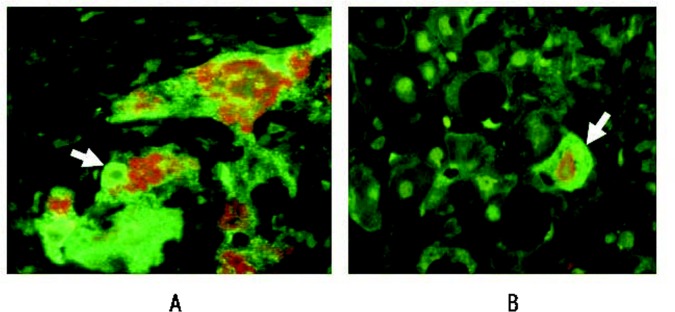
Immunohistochemical analysis of a liver biopsy obtained from a patient with alcoholic hepatitis with Mallory-Denk body (MDB) formation. The samples were stained for the presence of CD49f (integrin subunit α6) (green) and ubiquitin (red). Note that the MDBs stain both red for ubiquitin and green for CD49f. The arrows point to the nuclei that stain green except for the nucleolus. The yellow fringe on the MDB indicates colocalization of both proteins at the interface of the MDBs. The round black holes are macrovesicular fat globules in the hepatocytes. **A)** (magnification ×700) shows a cluster of MDB-forming cells. **B)** (magnification ×1,050) shows a single cell forming an MDB.

Machida and colleagues (2012) also found that TISCs isolated from the livers of alcohol-fed HCV Ns5a transgenic mice and from alcoholic patients carried molecules called CD133 and CD49f (i.e., were CD133^+^/CD49f^+^ cells). CD49f enhances the cell’s ability to differentiate into different cell types (i.e., multipotency) and maintains the cells’ stem-cell–like characteristics by directly controlling the regulatory molecules OCT4 and SOX2 (Yu et al. 2012). In addition, CD49f activates a signaling pathway called the phosphatidylinositol 3-kinase (P13K) AKT pathway and suppresses the levels of a protein called p53, which regulates the cell cycle and acts to prevent tumor formation (i.e., is a tumor suppressor gene). Immunohistochemical analyses of liver biopsies from patients with alcoholic hepatitis that contained numerous MDBs found that these cells expressed high levels of CD49f in the cytoplasm and the nuclei (see figure 5). This finding supports the concept that MDB-forming hepatocytes have progenitor and pluripotential properties and eventually may transform into TISC cells. Furthermore, in mice that were fed DCC and subsequently developed MDBs and, ultimately, HCC, CD49f, in combination with other molecules, induced MDB formation. This process could be blocked by inhibiting the phosphorylation of ERK and thus the activation of this protein as well as MDB formation (Wu et al. 2005).

## What About MicroRNAs?

MicroRNAs (miRNAs) are a class of small noncoding RNAs that, in general, negatively regulate gene expression at the posttranscriptional level. Each miRNA controls a specific set of target genes. miRNAs have been identified in various tumor types, including HCCs. miRNAs also are encoded by specific genes in the DNA. miRNA genes that harbor CpG islands can undergo methylation-mediated silencing, similar to many tumor suppressor genes. As a result, the miRNAs are not produced and therefore cannot inhibit the expression of their target genes. In one study examining the expression of 11 miRNA genes in HCCs, three of those genes were silenced (i.e., those encoding miRNAs miR-124, miR-203, and miR-375) (Furuta et al. 2010). For miR-124 and miR-203, the methylation frequently was tumor specific and was not found in nontumor tissue. Thus, these miRNAs were suppressive miRNAs for HCC that could be silenced epigenetically. This silencing resulted in the activation of multiple target genes (i.e., those encoding CDK6, vimentin, SET, and MYNO domain) (Furuta et al. 2010). Conversely, for other miRNAs the levels were increased in HCC, including miR-21, miR-34a, miR-221/222, miR-224, miR-106a, miR-92, miR-17-5 p, miR-20, and miR-18 (Bracconi and Patel 2008; Murakami et al. 2006). Finally, one miRNA (i.e., miR-126) was specific to HCC and alcohol use (Ladeiro et al. 2008).

Studies have shown that alcohol use regulates miRNAs that control transcriptional events and the expression of genes important to ALD (Mandrekar 2011). Mice fed alcohol as part of a liquid diet showed decreases in the levels of 1 percent of the total known miRNAs and increases in the levels of 3 percent of the miRNAs (Dolaniuc et al. 2009). For example, the levels of miR-182, miR-183, and miR-199a-3P were decreased, whereas those of miR-705 and miR-122 were increased. So far the miRNAs associated with HCC do not overlap with those associated with experimental ALD. However, there is overlap of changes in miRNA expression observed in mice fed a methyl-deficient diet for 12 weeks and those identified in HCCs (i.e., in both models the levels of miR-34a and miR-122 are changed) (Pogribny et al. 2009). In the methyl-deficient mice these miRNAs were associated with more extensive liver damage. These data mechanistically link alterations in microRNA expression to the pathogenesis of HCC and strongly suggest that differences in the susceptibility to liver carcinogenesis may be determined by differences in the miRNA expression response to factors such as methyl deficiency.

## Summary

ALD is a major cause of HCC, which usually develops long after alcohol abuse has ceased and when cirrhosis has developed. This clinical pattern suggests that changes in epigenetic liver cellular memory occur that affect differentiation and cellular renewal, as well as the transformation to HCC. Progenitor hepatocytes develop during the cirrhotic process from normal cells through epigenetic mechanisms, such as changes in DNA hyper- or hypomethylation, histone acetylation and methylation, and epigenetic reprogramming. For example, oxidative DNA damage from ethanol-induced ROSs leads to loss of methylated DNA. Chronic ethanol feeding leads to altered methionine metabolism and reduced DNA methylation because the levels of the major methyl donor SAMe are lowered. This process can be prevented by feeding SAMe or another compound called betaine together with ethanol.

Likewise, chronic ethanol feeding alters the methylation and acetylation of histones in the liver. Histone acetylation leads to upregulation of p21, causing cell cycle arrest and DNA damage. This, in turn, results in loss of DNA methylation, as demonstrated in experimental rat models as well as in human alcoholic hepatitis and HCCs. Histone H3K27me3, together with EZH2, regulates stem cell renewal and differentiation of progenitor stem cells in the liver. The balloon cells, which form MDBs in alcoholic hepatitis and HCCs, show a decrease in nuclear H3K27me3 and an increase in pEZH2 in the MDBs. This supports the role of MDB-forming cells as progenitor cells that give rise to HCC transformation. This concept is supported by findings that the MDB-forming cells also express proteins that are markers of embryonal stem cells (i.e., SOX2 and CD49f). The transformation of these progenitor cells to HCC is driven by the TLR4 signaling pathway, which is upregulated by increases in LPS levels in the liver that result from alcohol abuse. This upregulation of the TLR4 pathway, which has been demonstrated in rats chronically fed ethanol, can be prevented by SAMe supplementation. These and other findings support the concept that TLR4 may be a protooncogene responsible for the transformation of progenitor cells into HCC in ALD as well as HCV infection.

## Figures and Tables

**Table 1:** Abbreviations of the Protein Names Mentioned in This Article, Their Full Names, and Their Main Functions

**Acronym**	**Full Name**	**Function**
DNACD133	Prominin 1	Cancer stem cell marker
CD49f	Integrin α6	Cell adhesion
		Cell signaling
ERK	Extracellular signal-regulated kinase	Signaling pathway for growth of cells
EZH2	Enhancer of Zeste homology 2	Methylates DNA
MyD88	Myeloid differentiation response gene	Activates NFκB
Nanog	Named after Tir NanOg legend	Stem cell renewal
Oct 4	Octamer-binding transcription factor 4	Self-renewal of embryonal cells
p21 Waf1/C.p1	Type of p21 Cip/kip family	Regulates the cell cycle
p27	Type of p27 member Cip/kip	Regulates the cell cycle
pERK	Phosphorylated ERK	Activated ERK
Phospho AKT	AKT-mouse forming thymomas	Regulates cell survival
Threonine 308		
Phospho cJun	Early response transcription factor	Activates cJun
		Stimulates cell growth
Phospho-SAPK/JNK	Stress-activated protein kinase	Activates fetal liver formation
	Jun-amino kinase	
PPARPGC1α	PPAR γ coactivator 1 α	Regulates energy metabolism
PPARγ	Peroxisome proliferator-activated receptor γ	Regulates fatty acid storage
RARβ	Retinoic acid receptor β	Regulates cellular growth
SOX 2	SRY (Sex determination region Y) box 2	Induces pluripotential cells
TLR4	Toll-like receptor 4	Innate immunity
β catenin	Cadherin associated protein	Wnt signaling pathway

## Footnotes

1 Among other functions, the WNT/β-catenin pathway is involved in the fate of stem cells and regulates whether stem cells proliferate or self-renew. Accordingly, there is a strong correlation between WNT/β-catenin signaling and the onset of cancer. Normally, β-catenin cannot travel to the nucleus and is degraded. Under certain conditions, however, the degradation of β- catenin is prevented and it enters the nucleus. This can lead to excessive stem cell renewal and proliferation, predisposing the cells to the formation of tumors.

2 Direct mechanisms would include HAT p300-induced histone acetylation as seen in human HCC cells, whereas indirect mechanisms could include the induction of a molecule called integrin β17 through regulatory elements called Sp1 sites (Fang et al. 2007).

3 MDBs are inclusions found in the cytoplasm of liver cells and are indicative of liver damage; they are most commonly found in patients with ALD.

4 The term histone code refers to the hypothesis that modifications of different histones have different effects on gene expression—that is, whereas methylation of some histones activates gene expression, methylation of other histones may inhibit it. It is thought that the histone modifications serve to recruit other regulatory proteins that specifically recognize the modified histones. These recruited proteins then act to alter chromatin structure actively or to promote transcription. Enzymes that generate the histone modifications that make up this histone code are referred to as code writers.
